# Global research on cysticercosis and neurocysticercosis: A bibliometric analysis

**DOI:** 10.3389/fvets.2023.1156834

**Published:** 2023-04-11

**Authors:** Gregorio Gonzalez-Alcaide, Nestor Sosa, Laura Shevy, Isabel Belinchon-Romero, Jose-Manuel Ramos-Rincon

**Affiliations:** ^1^Department of History of Science and Documentation, University of Valencia, Valencia, Spain; ^2^Infectious Diseases Division, Internal Medicine Department, New Mexico University Health Sciences Center, Albuquerque, NM, United States; ^3^Department of Clinical Medicine, Miguel Hernández University, and Alicante Institute for Health and Biomedical Research (ISABIAL), Alicante, Spain

**Keywords:** cysticercosis, neurocysticercosis, *Tenia solium*, animals, human, bibliometrics

## Abstract

**Background:**

Cysticercosis is a parasitic infection caused by the larval stage *Taenia solium*. As a neglected tropical disease that is also difficult to diagnose, cysticercosis constitutes an important public health and research challenge. To characterize the development of research on cysticercosis and neurocysticercosis, considering the level of scientific evidence provided and the contribution of different countries to research, according to their endemic nature and their income level.

**Methods:**

Indexed publications on cysticercosis and neurocysticercosis were retrieved from the MEDLINE database, and the evolution of scientific production and the topic areas addressed in the body of research were analyzed.

**Results:**

A total of 7,860 papers published between 1928 and 2021 were analyzed. The volume of annual publications increased over time, standing at over 200 documents/year since 2010. Case studies constitute the main study design (27.4% of the documents with available information, *n* = 2,155), with fewer studies that provide the highest levels of scientific evidence, such as clinical studies (1.9%, *n* = 149) or systematic reviews (0.8%, *n* = 63). The most productive journals belong to the Parasitology and Tropical Medicine categories. Although the USA is the most productive country (*n* = 2,292), countries where *Tenia solium* is endemic, such as India (*n* = 1,749), Brazil (*n* = 941) and Peru (*n* = 898) also stand out, as does Mexico (*n* = 1,414). However, other endemic countries in Latin America and sub-Saharan Africa show little participation in the research. The level of international collaboration by country is very uneven, with some countries presenting very low values, such as India (9.9% of documents in international collaboration) or Brazil (18.7%); while there is evidence of intense international collaboration in countries like Peru (91.3%), Tanzania (88.2%) or Kenya (93.1%). Research output has coalesced in three thematic clusters: basic research in animals; parasitism, animal health, and zoonoses; and the diagnosis and therapeutic approach in diseases associated with cysticercosis and neurocysticercosis.

**Conclusions:**

The generation of knowledge on cysticercosis presents different features from other areas of research, such as the outstanding contribution of only some endemic countries; and the relevance of comprehensive approaches to research (animal and human health). Studies that provide higher levels of scientific evidence should be promoted, as should research in endemic areas.

## Introduction

Neglected tropical diseases (NTDs) are a heterogeneous group, defined by their prevalent nature in tropical or subtropical areas and their disproportional impact on impoverished communities, for which they impose a significant human, social and economic burden (1–3).

NTDs mainly comprise parasitic infections, such as Chagas disease, dracunculiasis, echinococcosis, trypanosomiasis, leishmaniasis, lymphatic filariasis, onchocerciasis, or taeniasis/cysticercosis. These diseases pose a major challenge due to their endemic nature in countries with relatively underdeveloped health systems and limited scientific capacity. Thus, research must prioritize the burdens and healthcare needs associated with the disease, which frequently encompass its proper diagnosis and assured treatment for the affected populations (4, 5).

Cysticercosis is caused by the larval stage of cestode *T. solium*. It is acquired by the ingestion of *T. solium* eggs, shed in the stool of a human tapeworm carrier. Following ingestion, embryos (oncospheres) hatch in the small intestine, invade the bowel wall, and disseminate hematogenously to brain (neurocysticercosis, causing different neurological symptoms like “epilepsy”) “striated muscles,” “liver,” and/or other tissues.

Neurocysticercosis is endemic to much of Asia, sub-Saharan Africa, and Latin America, where it constitutes a prominent public health problem associated with poverty and poor sanitary and hygienic conditions (6). Its prevalence is probably underestimated due both to the difficulty in diagnosing it (since it often produces mild and non-specific symptoms) and the lack of observational studies in different populations and countries (7). The estimated burden of human cysticercosis, according to systematic reviews or similar studies published from 2010 to 2015, is 2.78 million disability-adjusted life years (DALYs) (95% uncertainty intervals 2.14-3.61 million) (8). Neurocysticercosis is responsible for 30% to 70% of epilepsies in some endemic countries and regions (9, 10).

The literature contains extensive bibliometric analyses of NTDs such as leishmaniasis, Chagas, schistosomiasis, trypanosomiasis, and echinococcosis, among others, including some papers that focus on specific regions or countries such as Nigeria and India (11–15). However, no publications are available that analyze the scientific production on cysticercosis/neurocysticercosis.

The objective of this study is to describe the research on cysticercosis and neurocysticercosis, analyzing the evolution of scientific production, the distribution of contributions from different countries based on the endemicity of the disease and income level of the country, the relevance of international collaboration, and the defining features that characterize research on this NTD at the thematic and methodological level.

## Materials and methods

The MEDLINE database was used to carry out the analysis since it has the Medical Subject Headings (MeSH) thesaurus that allows very precise searches and because it is the most widely used source of information in the field of health sciences (mainly, English).

### Bibliographic searches

To identify the body of documents under study, two search processes were combined: the documents indexed using the MeSH term “Cysticercosis;” and a search with free-text terms.

### MeSH search

The MeSH thesaurus defines cysticercosis as “Infection with CYSTICERCUS, the larval form of the various tapeworms of the genus *Taenia* (usually *T. solium* in man). In humans, they penetrate the intestinal wall and invade subcutaneous tissue, brain, eye, muscle, heart, liver, lung, and peritoneum. Brain involvement results in NEUROCYSTICERCOSIS.” This last term has also been included in the MeSH since 1999 as a specific descriptor for cysticercosis, so by using the “explode” function, all the documents tagged with the neurocysticercosis descriptor were identified to characterize their content. The MeSH defines neurocysticercosis as “Infection of the brain, spinal cord, or perimeningeal structures with the larval forms of the genus *Taenia*“ (primarily *T. solium* in humans). Lesions formed by the organism are known as cysticerci. The infection may be subacute or chronic, and the severity of symptoms depends on the severity of the host immune response and the location and number of lesions. Seizures represent the most common clinical manifestation, although focal neurologic deficits may occur.

### Search with free-text terms

Likewise, two search strings were developed that consider all the possible synonyms of the terms “cysticercosis” and “neurocysticercosis” collected in the MeSH that could have been used in the title and abstract of the documents indexed in the MEDLINE database:

Cysticercosis[Title/Abstract] OR Cysticercoses[Title/Abstract] OR “Coenuri Infection^*^”[Title/Abstract] OR Coenurosis[Title/Abstract] OR Coenuroses[Title/Abstract] OR “Coenurus cerebralis Infection^*^”[Title/Abstract] OR “Cysticercus cellulosae Infection^*^”[Title/Abstract] OR “*T. solium* Cysticercosis”[Title/Abstract] OR “*T. solium* Cysticercoses”[Title/Abstract].

Neurocysticercosis[Title/Abstract] OR Neurocysticercoses[Title/Abstract] OR Neurocoenurosis[Title/Abstract] OR Neurocoenuroses[Title/Abstract] OR “Central Nervous System Cysticercosis”[Title/Abstract] OR “CNS Cysticercosis”[Title/Abstract] OR “CNS Cysticercoses”[Title/Abstract] OR “Brain Cysticercosis”[Title/Abstract] OR “Cerebral Coenurosis”[Title/Abstract] OR “Cerebral Coenuroses”[Title/Abstract] OR “Cerebral Cysticercosis”[Title/Abstract] OR “Cerebral Cysticercoses”[Title/Abstract].

The use of a thesaurus offers great consistency with regard to the precise identification of the relevant literature in relation to the topic under study, while using free-text terms favors the exhaustiveness of the searches, correcting possible gaps in the allocation processes of the MeSH terminology and, above all, making it possible to identify the most recent literature that has not yet been assigned descriptors. The MeSH thesaurus includes the term coenurosis as a synonym for cysticercosis and the terms neurocoenurosis/cerebral coenurosis as a synonym for neurocysticercosis. For this reason, both terms have been included in the search process. However, under current understanding among global parasitologists, cysticercus/cysticercosis is used for the larval stage of *T*. *solium*, coenurus/coenurosis is used for the larval stage of *T. multiceps* and *T. serialis*.

The searches were carried out in October 2022.

### Treatment of the bibliographic data

Bibliographic information from the identified documents was downloaded to review and standardize the data for their subsequent quantitative analysis. In this sense, the duplicated information from some fields (such as the MeSH descriptors) was individualized. The following processes were performed.

#### Document types

In addition to the formal classification of documents used widely by scientific journals (articles, reviews, and letters), the document categories identified in MEDLINE that are relevant from the clinical or epidemiological point of view were identified based on the research methodology used and the level of scientific evidence provided: case reports, clinical studies (controlled clinical trials/randomized controlled trials), observational studies, comparative studies, evaluation studies, guidelines/practice guidelines, systematic reviews, meta-analyses, and validation studies. Some documents were not assigned to any category, while others were assigned to more than one.

#### MeSH descriptors

The frequency of the MeSH terms assigned to the documents was determined, and a map of thematic clusters was generated, in order to obtain a visual overview of the thematic groupings comprising the body of research and the interrelationships established between the descriptors. For this purpose, a matrix was generated, quantifying how frequently the MeSH terms appeared together on the documents, and VOSViewer clustering algorithm was applied (https://www.vosviewer.com). Results are presented visually with VoSViewer. The clusters were labeled with concepts that broadly described the topic area of the MeSH terms included under each. The analysis was performed on 91.1% (*n* = 7,161) of the identified records that were assigned with the “cysticercosis” or “neurocysticercosis” descriptor.

#### Countries

For the analysis of the scientific output by country and the international collaboration, we used the documents identified in MEDLINE that were also collected in the Web of Science-Core Collection (WoS-CC), since this database includes information on institutional affiliations throughout the entire period analyzed, something that MEDLINE has only done systematically since 2014. Altogether, 68.8% (*n* = 5,411) of the documents analyzed are included in the WoS-CC, although in recent years around 85% to 90% are included. In addition to individualizing the instances with more than one institution to determine the different types of collaboration (domestic and international), the documents assigned to England, Scotland, Wales, and Northern Ireland were unified into the United Kingdom (UK). Domestic collaboration was defined as documents with authors from two or more institutions, administrative units, or departments of an institution from the same country. Authors from two or more institutions from different countries indicated international collaboration. In the case of documents with international collaboration, we also analyzed the country of the first author and corresponding author, roles that can be associated with research leadership, that is, a more relevant contribution to the conception, promotion, and responsibility for the studies carried out.

### Additional information and calculation of indicators

Records were also analyzed in terms of the endemicity of *T. solium* in the countries appearing in the institutional affiliations. Endemicity was determined using information from the World Health Organization (WHO) for 2022 (https://www.who.int/data/gho/data/indicators/indicator-details/GHO/status-of-endemicity-of-taenia-solium):

- Endemic countries;
- Suspected endemic;
- Few pigs with risk factors;
- Possible transmission in some communities;
- Non-endemic.

Scientific output was assessed according to the countries' economic development, as estimated from the gross national income (GNI) per capita, PPP (current international $), for 2021, using data provided by the International Comparison Program of the World Bank (https://data.worldbank.org/indicator/NY.GNP.PCAP.PP.CD), which classifies countries into:

-Low income;
-Lower middle income;
-Upper middle income;
-High income.

Once these processes were carried out, the following indicators were calculated:

#### Scientific production

-*N* documents per year of publication;
-*N* documents by document type and study design;
-*N* documents per journal;
-*N* documents per country.

#### Collaboration and leadership

-*N* documents in domestic collaboration;
-*N* documents in international collaboration;
-*N* documents as first signatories;
-*N* documents as corresponding authors.

#### Thematic areas

-Frequency of the MeSH terms assigned to the documents. The following categories were specifically identified and analyzed: humans/animals; qualifiers; diagnostic processes and techniques; and age groups;
-Research clusters, as generated based on the rest of descriptors.

## Results

In the MeSH search, the term “cysticercosis” and/or “neurocysticercosis” was assigned to 6,448 documents between 1928 (oldest reference identified) and 2021, while the free text search yielded another 1,499 records, a total 7,947 documents. The terms coenurosis and/or neurocoenurosis/cerebral coenurosis are present in the title of 104 documents and the abstract of 96 documents (in total 148 different documents, only 1.88% of the total number of documents analyzed). Eighty-seven records were excluded: 49 editorials and 38 other document types. The final analysis included only 7,860 articles, reviews, and letters—the document types commonly used in bibliometric studies because of their relevance for research and their reporting of original knowledge.

**Figure 1:**
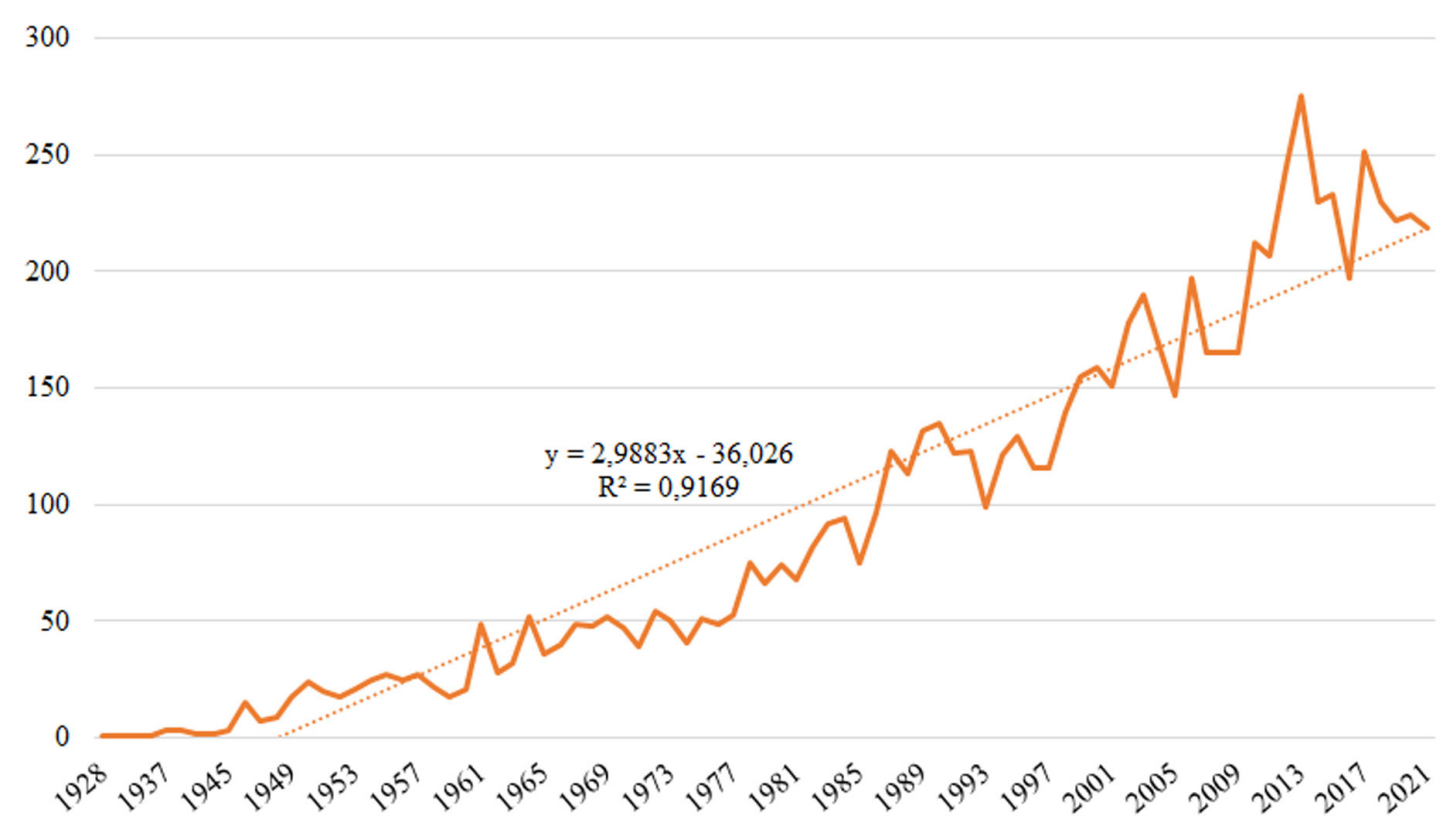
Evolution of the annual number of documents published on cysticercosis and neurocysticercosis and included in the MEDLINE database.

The main document type was journal article (*n* = 6,474; 82.4%), followed by review (*n* = 873, 11.1%) and letter (*n* = 513; 6.5%) (Table 1). Figure 1 shows the annual number of publications, which follows a linear growth trend (*R*^2^ = 0.92). Fewer than 20 documents were published annually from 1928 to 1948, 20 to 49 documents from 1950 to 1969, 50 to 99 from 1970 to 1987, 100 to 200 from 1988 to 1999, and more than 200 documents from 2010 to 2021.

**Table 1:** Publications on cysticercosis and neurocysticercosis indexed in the MEDLINE database, according to document type and study design.

	***n***	**%**
**Document type (*****N*** = **7,860)**		
Article	6,474	82.4
Review	873	11.1
Letter	513	6.5
**Study design (information available on** ***N*** = **2,931)**		
Case reports	2,155	27.4
Comparative study	306	3.9
Clinical study	149	1.9
Evaluation study	105	1.3
Controlled clinical trial/randomized controlled trial	76	1.0
Systematic review	63	0.8
Meta-analysis	33	0.4
Observational study	18	0.2
Validation study	10	0.1
Guideline/practice guideline	6	0.1
Others*	10	0.1

^*^8 clinical conference papers and 2 consensus papers.

There were 2,931 documents (37.3% of the total) with specific information available on the study design or type of evidence provided. Case reports were the most common type of study (*n* = 2,155, 27.4%), whereas papers presenting the highest levels of scientific evidence, such as clinical studies (1.9%, *n* = 149) or systematic reviews (0.8%, *n* = 63), were quite sparse, and the presence of clinical practice guidelines was anecdotal (0.1%, *n* = 6).

The 7,860 papers about cysticercosis were published in 1,554 journals. There were 23 journals (1.5%) that published more than 49 papers; together, these journals were responsible for 25.6% of the total records. Another 767 journals (49.4%) published a single document on the topic. The leading journals were *Arquivos de Neuro-Psiquiatria* (the official journal of the Brazilian Academy of Neurology) and the *American Journal of Tropical Medicine and Hygiene* (the official journal of the American Society of Tropical Medicine and Hygiene); each published 188 documents (2.4%). *Veterinary Parasitology* (the official organ of the American Association of Veterinary Parasitologists, the European Veterinary Parasitology College, and the World Association for the Advancement of Veterinary Parasitology) also stood out with 148 documents (1.9%) (Table 2).

**Table 2:** Top journals publishing papers on cysticercosis and neurocysticercosis and indexed in MEDLINE.

**Journal**	***N*** **docs**	%^*^	**Journal citation reports, science edition (2021)—ranking and quartile**
Arquivos de Neuro-Psiquiatria	188	2.4	Neurosciences 243/275 Q4 Psychiatry 132/155 Q4
The American Journal of Tropical Medicine and Hygiene	188	2.4	Public, environmental and occupational health 95/210 Q2 Tropical medicine 7/24 Q2
Veterinary Parasitology	148	1.9	Veterinary sciences 23/145 Q1 Parasitology 17/39 Q2
PLoS Neglected Tropical Diseases	122	1.6	Tropical medicine 2/24 Q1 Parasitology 5/39 Q1
Zhongguo ji sheng chong xue yu ji sheng chong bing za zhi = Chinese Journal of Parasitology and Parasitic Diseases	102	1.3	–
Acta Tropica	101	1.3	Tropical medicine 9/24 Q2 Parasitology 14/39 Q2
Parasitology Research	99	1.3	Parasitology 20/39 Q3
Transactions of the Royal Society of Tropical Medicine and Hygiene	97	1.2	Public, environmental and occupational health 150/210 Q3 Tropical medicine 13/24 Q3
Neurology	94	1.2	Clinical neurology 9/212 Q1
The Journal of Parasitology	88	1.1	Parasitology 32/39 Q4
Neurology India	84	1.1	Neurosciences 252/275 Q4
The Southeast Asian Journal of Tropical Medicine and Public Health	81	1.0	Public, environmental and occupational health 210/210 Q4 Tropical medicine 24/24 Q4 Infectious diseases 95/95 Q4
The Journal of the Association of Physicians of India	72	0.9	–
BMJ Case Reports	66	0.8	Medicine, general and internal—ESCI
Journal of Helminthology	58	0.7	Zoology 64/176 Q2 Parasitology 26/39 Q2
Experimental Parasitology	57	0.7	Parasitology 24/39 Q3
Parasitology	55	0.7	Parasitology 13/39 Q2
Parasites and Vectors	53	0.7	Tropical medicine 3/24 Q1 Parasitology 8/39 Q1
Epilepsia	52	0.7	Clinical neurology 27/212 Q1
International Journal for Parasitology	52	0.7	Parasitology 6/39 Q1
Revista do Instituto de Medicina Tropical de São Paulo	51	0.7	Tropical medicine 15/24 Q3 Parasitology 22/39 Q3 Infectious diseases 80/95 Q4
Revista de Neurologia	50	0.6	Clinical neurology 198/212 Q4
The New England Journal of Medicine	50	0.6	Medicine, general and internal 1/329 Q1

ESCI, Emerging Source Citation Index.

* % of total 7,860 papers on the topic.

The most productive journals included 7 high-impact journals (Q1 according to the journal impact factor ranking in the 2021 edition of *Journal Citation Reports, JCR*), along with various others with less visibility (4 journals in Q2, 4 journals in Q3, 5 journals in Q4 and one journal as a candidate in the *Emerging Source Citation Index*). Only two of the most productive journals (*Chinese Journal of Parasitology and Parasitic Diseases* and *The Journal of the Association of Physicians of India*) are not included in the JCR.

**Figure 2:**
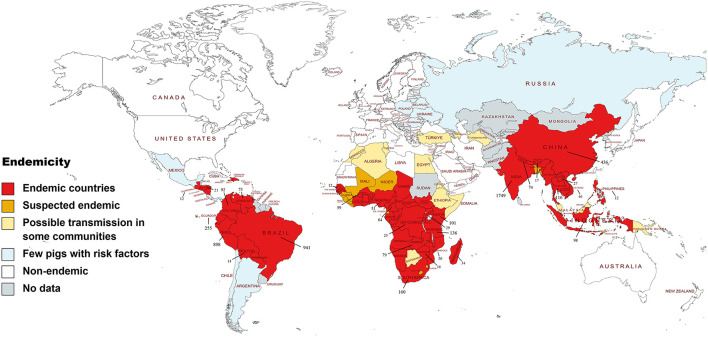
Research on cysticercosis and neurocysticercosis from countries where T. solium is endemic included in the MEDLINE database (> 10 papers).

Of the countries represented in the authors' institutional affiliations, 46.4% were non-endemic and 40.7%, endemic. The proportion of contributions by researchers from endemic countries increased from 39% in 1980–2000 to 69.4% in 2001–2021. In terms of economic development as measured using the GNI, authors in high-income countries participated in 47.7% of the documents, well ahead of participation by authors in upper middle-income (29.6%), lower middle-income (19.6%), and low-income countries (2.6%), even though endemic countries are concentrated in these latter categories (Table 3). Figure 2 shows research activity in endemic countries and Supplementary Annex I contains the complete list of scientific activity in endemic countries and countries where transmission is suspected.

**Table 3:** Participation in research on cysticercosis and neurocysticercosis by country, according to endemicity and income level (*N* = 6,972 publications included in the Web of Science Core Collection).

	**Up to 1979**	**% of 134**	**1980–2000**	**% of 1,074**	**2001–2021**	**% de 3,439**	**Total**	**% of 6,972**
**Endemicity for cysticercosis (*****n*** **countries; population in millions [M] or billions [B])**								
Endemic (55; 4.75 B)	28	20.9	419	39.0	2,388	69.4	2,835	40.7
Suspected endemic (10; 471 M)	0	0.0	0	0.0	4	0.1	4	0.1
Few pigs with risk factors (16; 427 M)	16	11.9	213	19.8	491	14.3	720	10.3
Possible transmission in some communities (13; 477 M)	3	2.2	8	0.7	70	2.0	81	1.2
Non-endemic (44; 1.26 B)	95	70.9	676	62.9	2,461	71.6	3,232	46.4
No data (17; 7 M)	0	0.0	22	2.1	78	2.3	100	1.4
**Income level (*****n*** **countries; population)**								
Low-income (15; 289 M)	1	0.8	19	1.8	160	4.7	180	2.6
Lower middle-income (27; 2.53 B)	19	14.2	165	15.4	1,185	34.5	1,369	19.6
Upper middle-income (13; 1.93 B)	19	14.2	449	41.8	1,597	46.4	2,065	29.6
High-income (0; 1.1 B)	103	76.9	688	64.1	2,531	73.6	3,322	47.7

In the analysis of scientific production by country, the most significant finding is that the highest production levels (>100 documents) were shared by countries endemic for *T. solium* along with some countries with the highest scientific development. Thus, after the USA (*n* = 2,292 documents), research output was highest in India (*n* = 1,749), Mexico (*n* = 1,414, non-endemic country, but classified as having “few pigs with risk factors”), Brazil (*n* = 941) and Peru (*n* = 898). Next, research was carried out in different countries in Europe and Asia (China, Japan, and South Korea), along with Australia, Canada, and other endemic countries such as Ecuador, Tanzania, Thailand, and Kenya (Table 4).

**Table 4:** Research production, collaboration, and leadership by country of institutional affiliations, in documents published on cysticercosis and neurocysticercosis and included in the Web of Science Core Collection.

**Country**	Endemicity^*^	Income level, GNI per capita^**^	***N*** **docs**	**Docs with domestic collaboration**		**Docs with intl collaboration**		**Docs with intl collaboration by 1**^**st**^ **author**		**Docs with intl collaboration by corresponding author**	
				***N***	**%**	***N***	**%**	***N***	**%**	***N***	**%**
USA	Non-endemic	High	2,292	1,812	79.1	1,162	50.7	197	17.0	211.15	18.2
India	Endemic	Lower middle	1,749	1,318	75.4	173	9.9	44	25.4	31.75	18.4
Mexico	Few pigs with risk factors	Upper middle	1,414	1,196	84.6	437	30.9	105	24.0	105.9	24.2
Brazil	Endemic	Upper middle	941	793	84.3	176	18.7	48	27.3	39.33	22.4
Peru	Endemic	Upper middle	898	858	95.6	820	91.3	119	14.5	115.5	14.0
UK	Non-endemic	High	511	285	55.8	399	78.1	75	18.8	71.57	17.9
China	Endemic	Upper middle	436	294	67.4	183	42.0	37	20.2	32.78	17.9
Belgium	Non-endemic	High	418	325	77.8	376	90.0	66	17.6	73.82	19.6
France	Non-endemic	High	368	291	79.1	151	41.0	26	17.2	28.41	18.8
Japan	Non-endemic	High	287	228	79.4	189	65.9	45	23.8	56.65	30.0
Spain	Non-endemic	High	287	199	69.3	99	34.5	17	17.2	15.75	15.9
S. Korea	Non-endemic	High	272	249	91.5	103	37.9	22	21.4	21	20.4
Australia	Non-endemic	High	268	158	59.0	130	48.5	38	29.2	38.24	29.4
Ecuador	Endemic	Upper middle	255	144	56.5	176	69.0	51	29.0	27.5	15.6
Germany	Non-endemic	High	236	170	72.0	138	58.5	27	19.6	19.08	13.8
Italy	Non-endemic	High	226	182	80.5	95	42.0	21	22.1	22.25	23.4
S. Africa	Endemic	Upper middle	160	99	61.9	87	54.4	17	19.5	14.33	16.5
Canada	Non-endemic	High	145	94	64.8	81	55.9	17	21.0	16	19.8
Denmark	Non-endemic	High	138	70	50.7	117	84.8	27	23.1	26.5	22.7
Tanzania	Endemic	Lower middle	136	99	72.8	120	88.2	21	17.5	19.5	16.3
Switzerland	Non-endemic	High	132	72	54.6	92	69.7	17	18.5	18.66	20.3
Thailand	Endemic	Upper middle	116	58	50.0	75	64.7	35	46.7	36.83	49.1
Kenya	Endemic	Lower middle	101	53	52.5	94	93.1	10	10.6	11.25	12.0

* Endemic status of T. solium.

Data source: World Health Organization, updated 31 Mar 2022. Available at: https://www.who.int/data/gho/data/indicators/indicator-details/GHO/status-of-endemicity-of-taenia-solium. ^**^Gross National Income (GNI) per capita, PPP (current international $), 2021 data, from International Comparison Program, World Bank. Available at: https://data.worldbank.org/indicator/NY.GNP.PCAP.PP.CD.

The manuscripts where there are authors from two or more institutions from different countries was considered an international collaboration (information available in 4,642 documents). Globally, 28.6% (*n* = 1,330) of the documents analyzed were produced in international collaboration. Collaboration increased throughout the study period, from 5.2% of the documents (*n* = 7) published before 1980, to 17.7% (*n* = 190) in 1980–2000, to 32.9% in 2001–2021.

In the case of documents with international collaboration, we also analyzed the country of the first author and corresponding author (information available in 4,642 documents), roles that can be associated with research leadership, that is, a more relevant contribution to the conception, promotion, and responsibility for the studies carried out The level of international collaboration varied widely by country, with some countries presenting very low values, such as India (9.9% of documents in international collaboration) or Brazil (18.7%); and others displaying high levels of collaboration, such as Peru (91.3%), Tanzania (88.2%), or Kenya (93.1%). However, this high international collaboration contrasts with their low levels of research leadership, with limited participation as first or corresponding authors (Table 4).

In relation to the thematic areas, research in animals shares the spotlight with research in humans, with both spheres coinciding more and more (Table 5). Overall, 46.9% of the documents (*n* = 3,690) are focused exclusively on research in humans compared to 16.8% in animals, although the documents assigned with both descriptors rose from 24.1% (*n* = 570) in 1980–2000 to 32.8% (*n* = 1,400) in 2001–2021.

**Table 5:** Documents on cysticercosis and neurocysticercosis assigned MeSH descriptors for research in humans and animals and indexed in MEDLINE.

**MeSH**	**Up to 1979 (*****N*** = **1,225)**		**1980–2000 (*****N*** = **2,365)**		**2001–2020 (*****N*** = **4,270)**		**Total**	
	***n***	**%**	***n***	**%**	***n***	**%**	***n***	**%**
Only humans	709	57.9	1,366	57.8	1,615	37.8	3,690	46.9
Only animals	263	21.5	373	15.8	682	16.0	1,318	16.8
Both	155	12.7	570	24.1	1,400	32.8	2,125	27.0

“Diagnosis” (*n* = 2,299) is the main qualifier assigned to the descriptors cysticercosis and neurocysticercosis, followed by “epidemiology” (*n* = 1,133), “complications” (*n* = 1,125) and “drug therapy” (*n* = 1,043). “Parasitology” (*n* = 1,019) and “veterinary” (*n* = 930) also have a notable presence (Table 6).

**Table 6:** Qualifiers accompanying the MeSH descriptors for cysticercosis and neurocysticercosis on documents indexed in MEDLINE.

**Qualifiers**	**Up to 1979 (*****N*** = **741)**		**1980–2000 (*****N*** = **2,036)**		**2001–2020 (*****N*** = **2,984)**		**Total**	
	***n***	**%**	***n***	**%**	***n***	**%**	***n***	**%**
Diagnosis	214	28.9	775	38.1	1,310	43.9	2,299	39.9
Epidemiology	130	17.5	297	14.6	706	23.7	1,133	19.7
Complications	106	14.3	356	17.5	663	22.2	1,125	19.5
Drug therapy	27	3.6	447	22.0	569	19.1	1,043	18.1
Parasitology	37	5.0	267	13.1	715	24.0	1,019	17.7
Veterinary	201	27.1	281	13.8	448	15.0	930	16.1
Pathology	137	18.5	282	13.9	480	16.1	899	15.6
Diagnostic imaging	59	8.0	316	15.5	370	12.4	745	12.9
Immunology	51	6.9	248	12.2	335	11.2	634	11.0
Surgery	79	10.7	170	8.4	238	8.0	487	8.5
Prevention and control	26	3.5	85	4.2	218	7.3	329	5.7
Therapy	9	1.2	82	4.0	159	5.3	250	4.3
Cerebrospinal fluid	22	3.0	121	5.9	72	2.4	215	3.7
Transmission	17	2.3	63	3.1	122	4.1	202	3.5
Physiopathology	10	1.4	52	2.6	137	4.6	199	3.5
Blood	4	0.5	47	2.3	119	4.0	170	3.0
Etiology	15	2.0	47	2.3	41	1.4	103	1.8

The most prominent descriptors related to diagnostic procedures and techniques were the diagnostic imaging procedures “tomography, x-ray computed” (*n* = 961), “magnetic resonance imaging” (*n* = 823) and, less frequently, “radiography” (*n* = 186) and “ultrasonography” (*n* = 86). Different methods and procedures for diagnosing diseases of the nervous system are also present, such as “electroencephalography” (*n* = 113) and “cerebral ventriculography” (*n* = 57).

Although “adult” (*n* = 2,315) is the main descriptor in relation to age groups, research linked to “adolescent” (*n* = 1,214) and “child” (*n* = 1,128) also has a notable presence (Table 7).

**Table 7:** MeSH descriptors related to age groups, assigned to scientific publications on cysticercosis and neurocysticercosis, indexed in MEDLINE.

**Age group**	**Up to 1979 (*****N*** = **1,225)**		**1980–2000 (*****N*** = **2,365)**		**2001–2020 (*****N*** = **4,270)**		**Total**	
	***n***	**%**	***n***	**%**	***n***	**%**	***n***	**%**
Adult	252	20.6	924	39.1	1,139	26.7	2,315	29.5
Aged, 80 and over	–	–	44	1.9	130	3.0	174	2.2
Middle Aged	155	12.7	626	26.5	762	17.9	1,543	19.6
Young Adult	1	0.1	–	–	386	9.0	387	4.9
Adolescent	92	7.5	489	20.7	633	14.8	1,214	15.5
Child	106	8.7	446	18.9	576	13.5	1,128	14.4
Child, Preschool	53	4.3	229	9.7	312	7.3	594	7.6
Infant	29	2.4	105	4.4	135	3.2	269	3.4
Infant, Newborn	8	0.7	27	1.1	48	1.1	83	1.1

**Figure 3:**
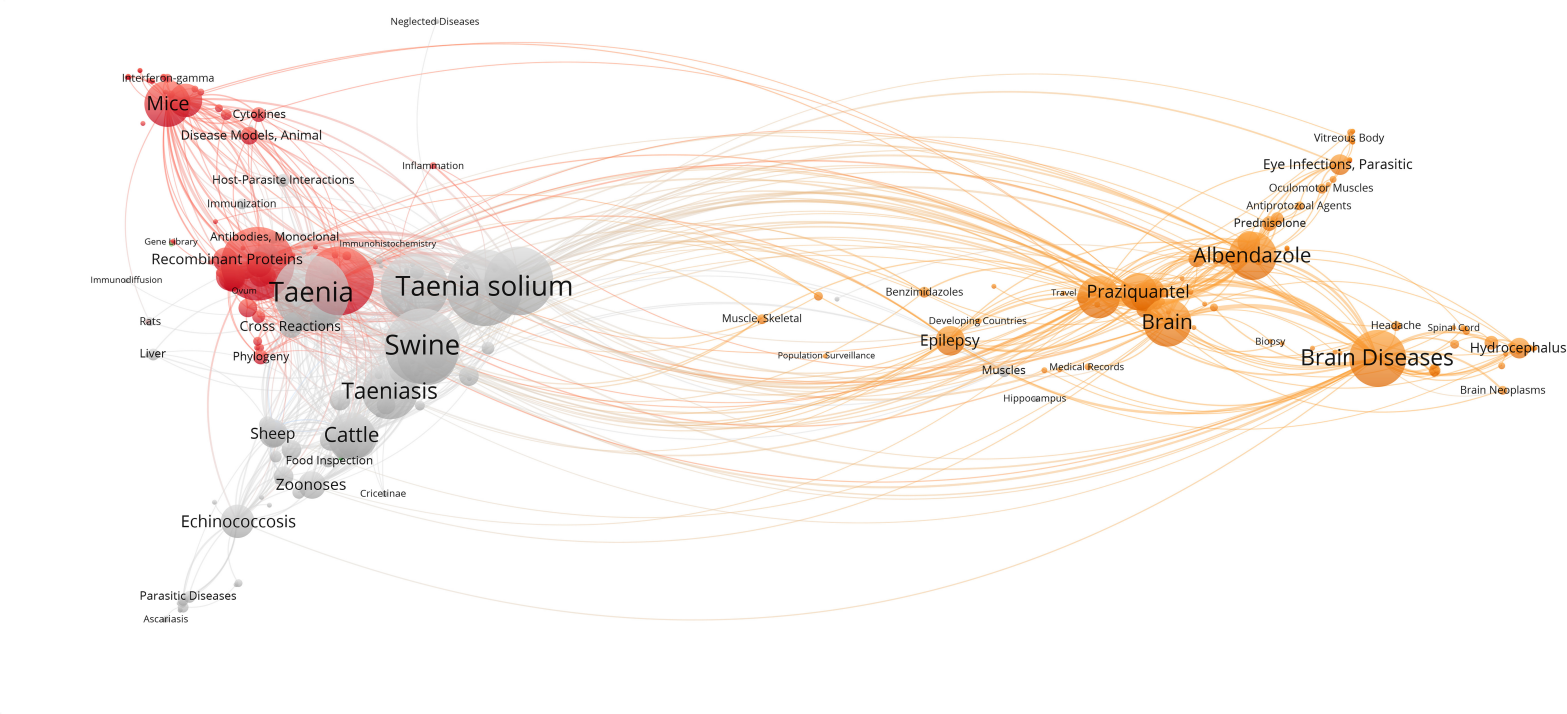
Thematic clusters generated from the MeSH descriptors assigned to the documents published on cysticercosis and neurocysticercosis and indexed in MEDLINE.

Figure 3 presents the network of thematic clusters generated from the co-occurrence of the MeSH terms. Research interest is concentrated in three spheres:

- **Basic research**. This cluster addresses the development of animal models, DNA sequencing, immunization, and vaccine development, among other aspects.

- **Parasitism, animal health and zoonoses**. Parasitic diseases linked to different animal species stand out in this cluster (echinococcosis, toxoplasmosis, trichinellosis, schistosomiasis, etc.). The possibility that the pathogens causing them are transmitted to humans (zoonosis) through food or inadequate hygienic conditions is reflected through different terms (e.g., health education, food health, rural health).

- **Diagnosis and therapeutic approach**. Within this area, brain diseases and eye diseases show specific development. There is a well-defined research cluster focusing on diseases that affect the brain, including epilepsy, hydrocephalus, brain neoplasms, gliomas, and meningitis. This cluster also encompasses associated symptoms and clinical manifestations, such as headache, intracranial pressure, and granulomas; and general descriptions of diagnostic methods, treatments (anti-inflammatory, antiparasitic, antiprotozoal or anthelmintic agents), and compounds (albenzadole, praziquantel, prednisone, prednisolone). Several eye diseases also stand out for the research interest they attract, including parasitic, conjunctive, retinal and orbital eye diseases.

Supplementary Annex II contains the list of the main MeSH terms (assigned to > 99 documents).

## Discussion

The evolution of scientific output on cysticercosis and neurocysticercosis shows a moderate but sustained increase in productivity in comparison to the study by Delgado et al. (16), who analyzed the publications indexed in MEDLINE from 1965 to 1995. Our results show that Latin American countries, together with the USA and Asian countries like India, China, Japan, and South Korea, lead research on the topic, while African countries are less present in the literature. These findings reflect a shift in scientific production since Delgado et al. (16) published their analysis, in which Asian and African researchers and institutions of reference were absent.

The scientific production on cysticercosis and neurocysticercosis in the present study (147–197 documents/year between 2001 and 2010 and over 200 documents per year since 2010) is significantly lower than that observed for other NTDs in MEDLINE, for example Chagas disease, with values above 500 documents per year since 2000 (17), or leishmaniasis, whose scientific output has been above 800 documents per year since the same date (18). There also seems to be more research activity on chikungunya, as the scientific output of the most recent year (2014) reported in the study by Vera-Polania et al. (19) was over 300 papers. This lower scientific production in global terms, together with other factors observed in this study, such as the lack of participation in research activities in many endemic countries or the fact that the damage associated with cysticercosis assessed using DALYs is higher than many other NTDs in recent published reports, including Chagas disease and leishmaniasis used as comparative references, highlights the relevance of promoting research in the field analyzed (20).

Research production on cysticercosis and neurocysticercosis is much greater in the USA than in Europe. The reasons for this difference reside in various factors, particularly the flow of immigrants from endemic countries to the USA (21, 22), which has led to the spread of neurocysticercosis to this country and its local transmission, making it a prominent public health problem. Researchers have highlighted the relevance of screening high-risk patients, prompt care for those infected, and transmission control, while the greater access to health and diagnostic systems in the USA compared to endemic countries also contributes to the research interest here.

The endemicity of the infection in the Indian subcontinent and in a large part of Latin America, areas where there is close contact with domestic animal hosts and less hygienic conditions (23–26), helps to explain the outstanding scientific production in countries such as India, Mexico, Brazil, and Peru, where there are prominent reference centers for research on the disease and its associated conditions, such as epilepsy (27, 28). In this regard, some studies have pointed out that infection is responsible for 30% of all epilepsies in developing countries (27, 28). On the other hand, other endemic countries in Latin America show little to no scientific production: no documents were authored by researchers in Paraguay, while the presence of other countries such as Haiti, the Dominican Republic, Nicaragua, and El Salvador is negligible. This is also the case for endemic countries in Africa: institutional affiliations in Togo, Rwanda, Gambia, the Republic of the Congo, Ghana, and Namibia are very limited, and no publications were identified with authors in Angola, Chad, South Sudan, Guinea-Bissau, or Cape Verde. The null participation in research on cysticercosis and neurocysticercosis also extends to “suspected endemic” countries (Bangladesh, Guinea, Sierra Leone, Lesotho, Equatorial Guinea, Eswatini, and Timor-Leste) or countries with “possible transmission” according to the WHO (Eritrea, Somalia and Turkmenistan). Furthermore, many other endemic countries have only made limited scientific contributions to the international literature (just 9 of the 55 endemic countries have published more than 100 papers). It is therefore essential to promote economic investments, structures, and collaborative initiatives to foster research in the geographical areas and low- to middle-income countries where the disease has the largest impact. Such initiatives are relevant both in the case of cysticercosis and neurocysticercosis, analyzed in the present study, and more broadly for the rest of the NTDs (29, 30).

The case of Peru is paradigmatic in terms of the development of biomedical research on cysticercosis and neurocysticercosis. There, policies aimed at promoting training programs and international alliances have nurtured the country's research culture and attracted international funding, which has resulted in a significant increase in scientific productivity, particularly on topics such as the most prevalent infectious diseases or endemic NTDs that affect the country (31).

Scientific collaboration has emerged as a crucial mechanism for promoting research and particularly for generating synergies between countries with more and less economic development. This has also been the experience in relation to other endemic as HIV infection or emerging NTDs as Chagas disease, that are difficult to control in countries with less socioeconomic development (12, 32, 33). In countries with lower income levels, smaller populations, and scientific systems with less capacity, including Peru but also Ecuador, Tanzania, Kenya and Thailand, the high level of international collaboration (65% to 93% of the documents on cysticercosis and neurocysticercosis) contrasts with the lower levels observed in larger countries like Mexico, Brazil, and India (< 31%). While international collaboration is positive, the fact that few researchers from low- to middle-income countries (e.g., Peru or Tanzania) take on leadership roles as first or corresponding authors shows the need to promote balance in research development, which involves strengthening the national scientific systems and scientific excellence in these settings (31, 34).

Research on cysticercosis and neurocysticercosis in humans is dominated by case studies (27.4% of publications vs. only 3.1% of clinical studies or 0.8% of systematic reviews). This can be explained by the fact that the disease has unusual manifestations, presents an atypical course, and requires differential treatments that need to be clearly communicated to the scientific community for clinical practice (35). Moreover, diagnosing neurocysticercosis is complex, especially in many endemic areas that lack the necessary diagnostic imaging equipment (36). For all these reasons, despite the fact that case studies are located at the bottom of the hierarchy of scientific evidence (37), this study design has outstanding relevance for diagnosis and clinical practice, as also determined in relation to other rare diseases and NTDs (38).

The fact that cysticercosis is a zoonotic disease, transmissible between animals and humans, reinforces the relevance of the One Health approach. This research paradigm integrates animal, human, and environmental health, and it favors mechanisms that allow the translation of evidence to health policies and practices (39). The impact of this approach on cysticercosis research is evident: the proportion of documents assigned with descriptors for both “human” and “animal” has increased from just 13% of those published before 1979 to 36% from the year 2000 onward. Furthermore, there is a notable presence of journals focused on tropical medicine in relation to human health (*American Journal of Tropical Medicine and Hygiene*); parasitology journals that cover human and animal health (particularly in the tropics and sub-tropics), the control of parasitic diseases, and the relationship between the host and the parasite (*Acta Tropica* or *Parasitology Research*); and veterinary journals, more focused on animal health, but whose research is crucial for global health (*Veterinary Parasitology*).

The thematic analysis enabled the identification and characterization of the main lines of inquiry that have shaped the development of research on cysticercosis and neurocysticercosis:

- *Basic research* and *animal models* allow a better understanding of the pathophysiology of the disease, determining the processes that take place during the development of the infection in the different species of animals and their immune response. As described in the literature, basic research in animals can contribute to determining risk factors and identifying specific biomarkers that signal the development of the disease or inform the development of vaccines (40, 41).

- The *transmission models of the T. solium parasite and the animal health control measures* represent tools for controlling the disease in humans, as reflected through different works that address food control, education, and health in rural areas, particularly in countries with low resources where this parasitism has a significant economic impact (42).

- Research on *the disease in humans, its prognosis and therapeutic approach*, constitute the third main research focus on cysticercosis and neurocysticercosis. Within this area, in relation to *diagnosis*, which remains the primary reference for research (16), research on brain diseases and parasitic eye diseases show specific development.

The fact that neurocysticercosis is one of the most common causes of epilepsy and other neurological manifestations, especially in endemic areas, is reflected in clinical research on brain diseases (9, 10, 43). The analysis also shows the relevance of the manifestations of cysticercosis outside the central nervous system, through ophthalmological involvement of the eyelids, the conjunctiva, and the anterior chamber (44), or the co-occurrence of intracranial and intraocular cysticercosis as a common phenomenon in clinical practice. Indeed, numerous cases of unusual presentations of cysticercosis in the eyes have also been published. Other organs that can be affected are the subcutaneous tissue, skeletal muscles, lungs, heart, thyroid, and pancreas (45).

Regarding the *therapeutic approach* to cysticercosis and neurocysticercosis, Singhi and Saini (46) highlighted the lack of a gold standard test to diagnose neurocysticercosis and the incipient nature of the management recommendations. Anthelmintics (drugs to treat worm infections) such as albendazole and praziquantel have been used to treat central nervous system lesions in neurocysticercosis, as have cysticides, although there is no clearly established clinical evidence (47). Thus, it is crucial to perform clinical trials that generate knowledge on adequate treatments (48, 49). Other aspects of treatment in humans relate to antiepileptic drugs to treat seizures (41) and the use of steroids, their indication, and duration (50, 51).

## Limitations

The main limitations of this study include the use of the MEDLINE database as a source of bibliographic data, which may have led to the omission of relevant publications in the analysis, particularly those published in endemic countries in languages other than English and literature related to psychosocial aspects. As an example, the same search carried out in the databases of the Virtual Health Library (VHL, https://bvsalud.org/en/), excluding MEDLINE, identifies 1,438 (18.09%) additional documents, with Spanish (*n* = 555, 6.98%), Portuguese (*n* = 298, 3.75%), Korean (*n* = 128, 1.61%) and Chinese (*n* = 77, 0.97%) standing out. Another relevant aspect to keep in mind is that the MeSH thesaurus includes the term coenurosis as a synonym for cysticercosis. This larval form, although present in members of the genus *Taenia*, is unrelated to *T. solium* or cysticercosis. This circumstance has had a limited incidence in the present analysis, may be relevant for future studies if these entities are not differentiated in the MeSH thesaurus. In addition, there are limitations inherent to the use of bibliometrics as a research methodology, which offers a quantitative vision of the development of research, as reflected in the form of publications and particularly scientific journals. Future lines of research can analyze the participation of research groups in publications on cysticercosis/neurocysticercosis and their impact.

## Conclusions

The generation of knowledge about cysticercosis as a neglected tropical disease presents different features from other areas of research. Case studies are the most prominent study design used, and some endemic countries are among the most prolific producers of research, in many cases overtaking the countries with far more established scientific systems. Nevertheless, authors from many other countries—especially those with less economic development—show negligible levels of participation. The relevance of comprehensive approaches to research (animal and human health) is also noteworthy. Studies that provide higher levels of scientific evidence should be promoted, and the participation and leadership of researchers from all endemic countries should be encouraged.

## Data availability statement

The raw data supporting the conclusions of this article will be made available by the authors, without undue reservation.

## Author contributions

J-MR-R and GG-A planned and designed the project, performed the analysis of data, and write original draft. NS, LS, and IB-R performed the interpretation of data. All authors reviewed the manuscript.
