# Supplementary material for: Global research on cysticercosis and neurocysticercosis: A bibliometric analysis
Source: Front Vet Sci. 2023 Apr 11;10:1156834. doi: 10.3389/fvets.2023.1156834 (PMC10126342; doi:10.3389/fvets.2023.1156834)
Supplement: Supplementary file 1 [file Table_1.DOCX]

Annex I. list of scientific activity in endemic countries and countries where transmission is suspected

| **Country** | **Endemicity*** | **GNI per capita** | **N documents** | **N documents DC** | **%** | **N documents IC** | **%** | **N documents IC 1^st^ author** | **%** | **N documents IC corresponding author** | **%** |
| --- | --- | --- | --- | --- | --- | --- | --- | --- | --- | --- | --- |
| India | Endemic | Lower middle income | 1749 | 1318 | 75,36 | 173 | 9,89 | 44 | 25,43 | 31,75 | 18,35 |
| Mexico | Few pigs with risk factors | Upper middle income | 1414 | 1196 | 84,58 | 437 | 30,91 | 105 | 24,03 | 105,9 | 24,23 |
| Brazil | Endemic | Upper middle income | 941 | 793 | 84,27 | 176 | 18,70 | 48 | 27,27 | 39,33 | 22,35 |
| Peru | Endemic | Upper middle income | 898 | 858 | 95,55 | 820 | 91,31 | 119 | 14,51 | 115,15 | 14,04 |
| Peoples R China | Endemic | Upper middle income | 436 | 294 | 67,43 | 183 | 41,97 | 37 | 20,22 | 0 | 0 |
| Ecuador | Endemic | Upper middle income | 255 | 144 | 56,47 | 176 | 69,02 | 51 | 28,98 | 27,5 | 15,63 |
| South Africa | Endemic | Upper middle income | 160 | 99 | 61,88 | 87 | 54,38 | 17 | 19,54 | 14,33 | 16,47 |
| Tanzania | Endemic | Lower middle income | 136 | 99 | 72,79 | 120 | 88,24 | 21 | 17,50 | 19,5 | 16,25 |
| Thailand | Endemic | Upper middle income | 116 | 58 | 50 | 75 | 64,66 | 35 | 46,67 | 36,83 | 49,11 |
| Kenya | Endemic | Lower middle income | 101 | 53 | 52,48 | 94 | 93,07 | 10 | 10,64 | 11,25 | 11,97 |
| Indonesia | Endemic | Lower middle income | 98 | 84 | 85,71 | 86 | 87,76 | 12 | 13,95 | 7,32 | 8,51 |
| Colombia | Endemic | Upper middle income | 92 | 72 | 78,26 | 51 | 55,43 | 11 | 21,57 | 8 | 15,69 |
| Zambia | Endemic | Low income | 79 | 54 | 68,35 | 76 | 96,20 | 14 | 18,42 | 10 | 13,16 |
| Nepal | Endemic | Lower middle income | 74 | 55 | 74,32 | 32 | 43,24 | 7 | 21,88 | 4,5 | 14,06 |
| Venezuela | Endemic | Upper middle income | 73 | 63 | 86,30 | 64 | 87,67 | 10 | 15,63 | 5,5 | 8,59 |
| Cameroon | Endemic | Lower middle income | 64 | 47 | 73,44 | 56 | 87,50 | 4 | 7,14 | 5 | 8,93 |
| Burkina Faso | Endemic | Low income | 59 | 56 | 94,92 | 52 | 88,14 | 5 | 9,62 | 4 | 7,69 |
| Turkey | Possible transmission in some communities | High income | 52 | 43 | 82,69 | 9 | 17,31 | 1 | 11,11 | 1 | 11,11 |
| Nigeria | Endemic | Lower middle income | 51 | 30 | 58,82 | 30 | 58,82 | 8 | 26,67 | 7 | 23,33 |
| Argentina | Few pigs with risk factors | Upper middle income | 47 | 30 | 63,83 | 34 | 72,34 | 4 | 11,76 | 4,66 | 13,71 |
| Vietnam | Endemic | Lower middle income | 46 | 30 | 65,22 | 31 | 67,39 | 8 | 25,81 | 5,83 | 18,81 |
| Egypt | Possible transmission in some communities | Lower middle income | 37 | 31 | 83,78 | 29 | 78,38 | 8 | 27,59 | 5,5 | 18,97 |
| Madagascar | Endemic | Low income | 34 | 25 | 73,53 | 22 | 64,71 | 5 | 22,73 | 2,33 | 10,59 |
| Chile | Few pigs with risk factors | High income | 34 | 23 | 67,65 | 8 | 23,53 | 2 | 25 | 2 | 25 |
| Ethiopia | Possible transmission in some communities | Low income | 31 | 24 | 77,42 | 11 | 35,48 | 4 | 36,36 | 3 | 27,27 |
| Mozambique | Endemic | Low income | 30 | 22 | 73,33 | 30 | 100 | 10 | 33,33 | 7,5 | 25 |
| Uganda | Endemic | Low income | 29 | 23 | 79,31 | 27 | 93,10 | 5 | 18,52 | 4 | 14,81 |
| Serbia | Few pigs with risk factors | Upper middle income | 29 | 14 | 48,28 | 15 | 51,72 | 1 | 6,67 | 1,33 | 8,87 |
| Malaysia | Possible transmission in some communities | Upper middle income | 29 | 16 | 55,17 | 14 | 48,28 | 3 | 21,43 | 4,5 | 32,14 |
| Poland | Few pigs with risk factors | High income | 28 | 17 | 60,71 | 7 | 25 | 2 | 28,57 | 1 | 14,29 |
| Laos | Endemic | Lower middle income | 27 | 22 | 81,48 | 27 | 100 | 0 | 0 | 1 | 3,70 |
| Croatia | Few pigs with risk factors | High income | 27 | 27 | 100 | 2 | 7,41 | 0 | 0 | 0 | 0 |
| DR Congo | Endemic | Low income | 25 | 21 | 84 | 21 | 84 | 2 | 9,52 | 1,33 | 6,33 |
| Honduras | Endemic | Lower middle income | 21 | 13 | 61,90 | 19 | 90,48 | 4 | 21,05 | 3 | 15,79 |
| Bhutan | Endemic | Lower middle income | 17 | 16 | 94,12 | 17 | 100 | 2 | 11,76 | 0,5 | 2,94 |
| Zimbabwe | Endemic | Lower middle income | 16 | 6 | 37,50 | 5 | 31,25 | 3 | 60 | 2 | 40 |
| Bolivia | Endemic | Lower middle income | 14 | 7 | 50 | 13 | 92,86 | 1 | 7,69 | 0 | 0 |
| Guatemala | Endemic | Upper middle income | 12 | 10 | 83,33 | 9 | 75 | 2 | 22,22 | 0 | 0 |
| Philippines | Endemic | Lower middle income | 12 | 3 | 25 | 11 | 91,67 | 3 | 27,27 | 2 | 18,18 |
| Senegal | Endemic | Lower middle income | 12 | 7 | 58,33 | 5 | 41,67 | 1 | 20 | 1 | 20 |
| Malawi | Endemic | Low income | 10 | 8 | 80 | 9 | 90 | 3 | 33,33 | 3 | 33,33 |
| Romania | Few pigs with risk factors | High income | 10 | 9 | 90 | 5 | 50 | 1 | 20 | 0 | 0 |
| Nicaragua | Endemic | Lower middle income | 9 | 4 | 44,44 | 9 | 100 | 0 | 0 | 0 | 0 |
| Cote Ivoire | Endemic | Lower middle income | 8 | 8 | 100 | 8 | 100 | 2 | 25 | 1,16 | 14,50 |
| Rwanda | Endemic | Low income | 7 | 3 | 42,86 | 7 | 100 | 1 | 14,29 | 2 | 28,57 |
| Togo | Endemic | Low income | 7 | 4 | 57,14 | 5 | 71,43 | 3 | 60 | 1 | 20 |
| Estonia | Few pigs with risk factors | High income | 7 | 5 | 71,43 | 7 | 100 | 2 | 28,57 | 1 | 14,29 |
| Burundi | Endemic | Low income | 6 | 2 | 33,33 | 6 | 100 | 3 | 50 | 1 | 16,67 |
| Russia | Few pigs with risk factors | Upper middle income | 6 | 6 | 100 | 6 | 100 | 0 | 0 | 0 | 0 |
| Myanmar | Endemic | Lower middle income | 5 | 2 | 40 | 5 | 100 | 0 | 0 | 0 | 0 |
| Namibia | Endemic | Upper middle income | 5 | 4 | 80 | 4 | 80 | 2 | 50 | 1 | 25 |
| Latvia | Few pigs with risk factors | High income | 5 | 4 | 80 | 4 | 80 | 0 | 0 | 0 | 0 |
| Panama | Few pigs with risk factors | High income | 5 | 4 | 80 | 5 | 100 | 1 | 20 | 1 | 20 |
| Oman | Non-endemic | High income | 5 | 3 | 60 | 1 | 20 | 0 | 0 | 0 | 0 |
| Benin | Endemic | Lower middle income | 4 | 4 | 100 | 4 | 100 | 1 | 25 | 1 | 25 |
| Cambodia | Endemic | Lower middle income | 4 | 2 | 50 | 4 | 100 | 0 | 0 | 0 | 0 |
| Gabon | Endemic | Upper middle income | 4 | 2 | 50 | 4 | 100 | 1 | 25 | 1 | 25 |
| Costa Rica | Few pigs with risk factors | Upper middle income | 4 | 2 | 50 | 2 | 50 | 0 | 0 | 0 | 0 |
| Central African Republic | Endemic | Low income | 3 | 3 | 100 | 3 | 100 | 0 | 0 | 0 | 0 |
| Gambia | Endemic | Low income | 3 | 2 | 66,67 | 3 | 100 | 0 | 0 | 0 | 0 |
| Ghana | Endemic | Lower middle income | 3 | 0 | 0 | 3 | 100 | 0 | 0 | 0 | 0 |
| Haiti | Endemic | Lower middle income | 3 | 2 | 66,67 | 3 | 100 | 0 | 0 | 0 | 0 |
| North Macedonia | Few pigs with risk factors | Upper middle income | 3 | 3 | 100 | 3 | 100 | 0 | 0 | 0 | 0 |
| Morocco | Possible transmission in some communities | Lower middle income | 3 | 2 | 66,67 | 0 | 0 | 0 | 0 | 0 | 0 |
| Bosnia & Herzegovina | Few pigs with risk factors | Upper middle income | 2 | 2 | 100 | 2 | 100 | 1 | 50 | 0 | 0 |
| Tunisia | Possible transmission in some communities | Lower middle income | 2 | 0 | 0 | 1 | 50 | 0 | 0 | 0 | 0 |
| Mali | Suspected endemic | Low income | 2 | 0 | 0 | 2 | 100 | 0 | 0 | 0 | 0 |
| Dominican Rep | Endemic | Upper middle income | 1 | 0 | 0 | 1 | 100 | 0 | 0 | 0 | 0 |
| El Salvador | Endemic | Lower middle income | 1 | 0 | 0 | 1 | 100 | 0 | 0 | 0 | 0 |
| Rep Congo | Endemic | Lower middle income | 1 | 0 | 0 | 1 | 100 | 0 | 0 | 0 | 0 |
| Rhodesia | Endemic | Lower middle income | 1 | 0 | 0 | 0 | 0 | 0 | 0 | 0 | 0 |
| Lithuania | Few pigs with risk factors | High income | 1 | 0 | 0 | 1 | 100 | 0 | 0 | 0 | 0 |
| Algeria | Possible transmission in some communities | Lower middle income | 1 | 0 | 0 | 1 | 100 | 0 | 0 | 0 | 0 |
| Botswana | Possible transmission in some communities | Upper middle income | 1 | 0 | 0 | 1 | 100 | 1 | 100 | 1 | 100 |
| Lebanon | Possible transmission in some communities | Lower middle income | 1 | 0 | 0 | 1 | 100 | 1 | 100 | 0 | 0 |
| Papua N Guinea | Possible transmission in some communities | Lower middle income | 1 | 0 | 0 | 1 | 100 | 0 | 0 | 0 | 0 |
| Liberia | Suspected endemic | Low income | 1 | 0 | 0 | 1 | 100 | 1 | 100 | 0 | 0 |
| Niger | Suspected endemic | Low income | 1 | 0 | 0 | 1 | 100 | 0 | 0 | 0 | 0 |
| Cabo Verde | Endemic | Lower middle income | 0 | 0 | 0 | 0 | 0 | 0 | 0 | 0 | 0 |
| Chad | Endemic | Low income | 0 | 0 | 0 | 0 | 0 | 0 | 0 | 0 | 0 |
| Guinea-Bissau | Endemic | Low income | 0 | 0 | 0 | 0 | 0 | 0 | 0 | 0 | 0 |
| Paraguay | Endemic | Upper middle income | 0 | 0 | 0 | 0 | 0 | 0 | 0 | 0 | 0 |
| South Sudan | Endemic | Low income | 0 | 0 | 0 | 0 | 0 | 0 | 0 | 0 | 0 |
| Eritrea | Possible transmission in some communities | Low income | 0 | 0 | 0 | 0 | 0 | 0 | 0 | 0 | 0 |
| Somalia | Possible transmission in some communities | Low income | 0 | 0 | 0 | 0 | 0 | 0 | 0 | 0 | 0 |
| Turkmenistan | Possible transmission in some communities | Upper middle income | 0 | 0 | 0 | 0 | 0 | 0 | 0 | 0 | 0 |
| Bangladesh | Suspected endemic | Lower middle income | 0 | 0 | 0 | 0 | 0 | 0 | 0 | 0 | 0 |
| Equatorial Guinea | Suspected endemic | Upper middle income | 0 | 0 | 0 | 0 | 0 | 0 | 0 | 0 | 0 |
| Eswaitini | Suspected endemic | Lower middle income | 0 | 0 | 0 | 0 | 0 | 0 | 0 | 0 | 0 |
| Guinea | Suspected endemic | Low income | 0 | 0 | 0 | 0 | 0 | 0 | 0 | 0 | 0 |
| Lesotho | Suspected endemic | Lower middle income | 0 | 0 | 0 | 0 | 0 | 0 | 0 | 0 | 0 |
| Sierra Leone | Suspected endemic | Low income | 0 | 0 | 0 | 0 | 0 | 0 | 0 | 0 | 0 |
| Timor-Leste | Suspected endemic | Lower middle income | 0 | 0 | 0 | 0 | 0 | 0 | 0 | 0 | 0 |
