# Supplementary material for: Global research on cysticercosis and neurocysticercosis: A bibliometric analysis
Source: Front Vet Sci. 2023 Apr 11;10:1156834. doi: 10.3389/fvets.2023.1156834 (PMC10126342; doi:10.3389/fvets.2023.1156834)
Supplement: Supplementary file 2 [file Table_2.DOCX]

Annex II- List of the main MeSH terms (assigned to > 99 documents).

| **MeSH Heading** | **Before 1980** | **%** | **1980-2000** | **%** | **2001-2021** | **%** | **Total** | **%** |
| --- | --- | --- | --- | --- | --- | --- | --- | --- |
| Brain Diseases | 358 | 29,22 | 750 | 31,71 | 177 | 4,15 | 1285 | 16,35 |
| Tomography, X-Ray Computed | 10 | 0,82 | 540 | 22,83 | 411 | 9,63 | 961 | 12,23 |
| Taenia solium | 9 | 0,73 | 1 | 0,04 | 932 | 21,83 | 942 | 11,98 |
| Taenia | 132 | 10,78 | 412 | 17,42 | 387 | 9,06 | 931 | 11,84 |
| Cysticercus | 83 | 6,78 | 375 | 15,86 | 428 | 10,02 | 886 | 11,27 |
| Magnetic Resonance Imaging | 0 | 0 | 225 | 9,51 | 598 | 14,00 | 823 | 10,47 |
| Swine | 56 | 4,57 | 203 | 8,58 | 523 | 12,25 | 782 | 9,95 |
| Brain | 192 | 15,67 | 185 | 7,82 | 364 | 8,52 | 741 | 9,43 |
| Enzyme-Linked Immunosorbent Assay | 5 | 0,41 | 266 | 11,25 | 414 | 9,70 | 685 | 8,72 |
| Antigens, Helminth | 0 | 0 | 242 | 10,23 | 421 | 9,86 | 663 | 8,44 |
| Antibodies, Helminth | 0 | 0 | 242 | 10,23 | 406 | 9,51 | 648 | 8,24 |
| Diagnosis, Differential | 56 | 4,57 | 204 | 8,63 | 355 | 8,31 | 615 | 7,82 |
| Taeniasis | 84 | 6,86 | 143 | 6,05 | 365 | 8,55 | 592 | 7,53 |
| Swine Diseases | 37 | 3,02 | 131 | 5,54 | 377 | 8,83 | 545 | 6,93 |
| Albendazole | 0 | 0 | 168 | 7,10 | 362 | 8,48 | 530 | 6,74 |
| Anthelmintics | 21 | 1,71 | 127 | 5,37 | 344 | 8,06 | 492 | 6,26 |
| Cattle | 161 | 13,14 | 149 | 6,30 | 165 | 3,86 | 475 | 6,04 |
| Praziquantel | 3 | 0,24 | 336 | 14,21 | 124 | 2,90 | 463 | 5,89 |
| Epilepsy | 43 | 3,51 | 137 | 5,79 | 265 | 6,21 | 445 | 5,66 |
| Prevalence | 1 | 0,08 | 96 | 4,06 | 323 | 7,56 | 420 | 5,34 |
| Cattle Diseases | 130 | 10,61 | 128 | 5,41 | 132 | 3,09 | 390 | 4,96 |
| Mice | 27 | 2,20 | 84 | 3,55 | 225 | 5,27 | 336 | 4,27 |
| Seizures | 8 | 0,65 | 66 | 2,79 | 238 | 5,57 | 312 | 3,97 |
| Echinococcosis | 75 | 6,12 | 114 | 4,82 | 105 | 2,46 | 294 | 3,74 |
| Treatment Outcome | 0 | 0 | 41 | 1,73 | 229 | 5,36 | 270 | 3,44 |
| Risk Factors | 0 | 0 | 37 | 1,56 | 181 | 4,24 | 218 | 2,77 |
| Zoonoses | 23 | 1,88 | 39 | 1,65 | 150 | 3,51 | 212 | 2,70 |
| Mice, Inbred BALB C | 0 | 0 | 44 | 1,86 | 167 | 3,91 | 211 | 2,68 |
| Sheep | 54 | 4,41 | 65 | 2,75 | 80 | 1,87 | 199 | 2,53 |
| Hydrocephalus | 23 | 1,88 | 78 | 3,30 | 97 | 2,27 | 198 | 2,52 |
| Eye Infections, Parasitic | 4 | 0,33 | 51 | 2,16 | 137 | 3,21 | 192 | 2,44 |
| Radiography | 64 | 5,22 | 40 | 1,69 | 82 | 1,92 | 186 | 2,37 |
| Cerebral Ventricles | 39 | 3,18 | 82 | 3,47 | 60 | 1,41 | 181 | 2,30 |
| Cestode Infections | 62 | 5,06 | 40 | 1,69 | 70 | 1,64 | 172 | 2,19 |
| Anticonvulsants | 1 | 0,08 | 30 | 1,27 | 140 | 3,28 | 171 | 2,18 |
| Meat | 37 | 3,02 | 43 | 1,82 | 91 | 2,13 | 171 | 2,18 |
| Calcinosis | 19 | 1,55 | 58 | 2,45 | 89 | 2,08 | 166 | 2,11 |
| Immunoglobulin G | 6 | 0,49 | 68 | 2,88 | 92 | 2,15 | 166 | 2,11 |
| Sheep Diseases | 42 | 3,43 | 54 | 2,28 | 68 | 1,59 | 164 | 2,09 |
| Serologic Tests | 17 | 1,39 | 49 | 2,07 | 97 | 2,27 | 163 | 2,07 |
| Immunoblotting | 0 | 0 | 59 | 2,49 | 94 | 2,20 | 153 | 1,95 |
| Central Nervous System Diseases | 25 | 2,04 | 110 | 4,65 | 7 | 0,16 | 142 | 1,81 |
| Seroepidemiologic Studies | 0 | 0 | 29 | 1,23 | 110 | 2,58 | 139 | 1,77 |
| Cysts | 13 | 1,06 | 44 | 1,86 | 82 | 1,92 | 139 | 1,77 |
| Feces | 12 | 0,98 | 43 | 1,82 | 83 | 1,94 | 138 | 1,76 |
| Drug Therapy, Combination | 1 | 0,08 | 49 | 2,07 | 86 | 2,01 | 136 | 1,73 |
| Eye Diseases | 70 | 5,71 | 35 | 1,48 | 26 | 0,61 | 131 | 1,67 |
| Brain Neoplasms | 23 | 1,88 | 52 | 2,20 | 55 | 1,29 | 130 | 1,65 |
| Taenia saginata | 14 | 1,14 | 1 | 0,04 | 114 | 2,67 | 129 | 1,64 |
| Anticestodal Agents | 1 | 0,08 | 29 | 1,23 | 97 | 2,27 | 127 | 1,62 |
| Helminth Proteins | 0 | 0 | 25 | 1,06 | 101 | 2,37 | 126 | 1,60 |
| Rural Population | 1 | 0,08 | 16 | 0,68 | 107 | 2,51 | 124 | 1,58 |
| Abattoirs | 24 | 1,96 | 21 | 0,89 | 78 | 1,83 | 123 | 1,56 |
| Blotting, Western | 0 | 0 | 52 | 2,20 | 71 | 1,66 | 123 | 1,56 |
| Dogs | 33 | 2,69 | 39 | 1,65 | 47 | 1,10 | 119 | 1,51 |
| Disease Models, Animal | 2 | 0,16 | 25 | 1,06 | 91 | 2,13 | 118 | 1,50 |
| Spinal Cord Diseases | 25 | 2,04 | 44 | 1,86 | 49 | 1,15 | 118 | 1,50 |
| Larva | 18 | 1,47 | 40 | 1,69 | 59 | 1,38 | 117 | 1,49 |
| Cross Reactions | 5 | 0,41 | 61 | 2,58 | 50 | 1,17 | 116 | 1,48 |
| Parasitic Diseases | 24 | 1,96 | 42 | 1,78 | 49 | 1,15 | 115 | 1,46 |
| Molecular Sequence Data | 0 | 0 | 29 | 1,23 | 84 | 1,97 | 113 | 1,44 |
| Electroencephalography | 18 | 1,47 | 41 | 1,73 | 54 | 1,26 | 113 | 1,44 |
| Time Factors | 14 | 1,14 | 45 | 1,90 | 53 | 1,24 | 112 | 1,42 |
| Incidence | 9 | 0,73 | 20 | 0,85 | 83 | 1,94 | 112 | 1,42 |
| Age Factors | 20 | 1,63 | 38 | 1,61 | 49 | 1,15 | 107 | 1,36 |
| Granuloma | 3 | 0,24 | 30 | 1,27 | 70 | 1,64 | 103 | 1,31 |
| Muscular Diseases | 45 | 3,67 | 26 | 1,10 | 32 | 0,75 | 103 | 1,31 |
| Amino Acid Sequence | 0 | 0 | 27 | 1,14 | 76 | 1,78 | 103 | 1,31 |
| Muscles | 47 | 3,84 | 39 | 1,65 | 16 | 0,37 | 102 | 1,30 |
| Host-Parasite Interactions | 2 | 0,16 | 30 | 1,27 | 70 | 1,64 | 102 | 1,30 |
| Rabbits | 26 | 2,12 | 25 | 1,06 | 51 | 1,19 | 102 | 1,30 |
| Meningitis | 32 | 2,61 | 35 | 1,48 | 34 | 0,80 | 101 | 1,28 |
| Animal Husbandry | 3 | 0,24 | 8 | 0,34 | 89 | 2,08 | 100 | 1,27 |
| Recombinant Proteins | 0 | 0 | 20 | 0,85 | 80 | 1,87 | 100 | 1,27 |
